# Zebrafish Microenvironment Elevates EMT and CSC-Like Phenotype of Engrafted Prostate Cancer Cells

**DOI:** 10.3390/cells9040797

**Published:** 2020-03-26

**Authors:** Lanpeng Chen, Maciej Boleslaw Olszewski, Marianna Kruithof-de Julio, B. Ewa Snaar-Jagalska

**Affiliations:** 1 Institute of Biology, Leiden University, 2311 EZ Leiden, Netherlands; l.chen@biology.leidenuniv.nl; 2 Department of Molecular Biology, International Institute of Molecular and Cell Biology, 02-109 Warsaw, Poland; maciej@olszewski.science; 3 Center for Biomedical Research, Institute of Veterinary Medicine, Warsaw University of Life Sciences, 02-787 Warsaw, Poland; 4 Department for BioMedical Research, Bern University, 3012 Bern, Switzerland; marianna.kruithofdejulio@dbmr.unibe.ch; 5 Department of Urology, Inselspital, 3008 Bern, Switzerland

**Keywords:** Zebrafish xenograft model, prostate cancer, cell plasticity

## Abstract

To visually and genetically trace single-cell dynamics of human prostate cancer (PCa) cells at the early stage of metastasis, a zebrafish (ZF) xenograft model was employed. The phenotypes of intravenously transplanted fluorescent cells were monitored by high-resolution, single-cell intravital confocal and light-sheet imaging. Engrafted osteotropic, androgen independent PCa cells were extravasated from caudle vein, invaded the neighboring tissue, proliferated and formed experimental metastases around caudal hematopoietic tissue (CHT) in four days. Gene expression comparison between cells in culture and in CHT revealed that engrafted PCa cells responded to the ZF microenvironment by elevating expression of epithelial–mesenchymal transition (EMT) and stemness markers. Next, metastatic potentials of ALDH^hi^ cancer stem-like cells (CSCs) and ALDH^low^ non-CSCs were analyzed in ZF. Engraftment of CSCs induced faster metastatic onset, however after six days both cell subpopulations equally responded to the ZF microenvironment, resulting in the same increase of stemness genes expression including Nanog, Oct-4 and Cripto. Knockdown of Cripto significantly reduced the vimentin/E-cadherin ratio in engrafted cells, indicating that Cripto is required for transduction of the microenvironment signals from the ZF niche to increase mesenchymal potential of cells. Targeting of either Cripto or EMT transcriptional factors Snail 1 and Zeb1 significantly suppressed metastatic growth. These data indicated that zebrafish microenvironment governed the CSC/EMT plasticity of human PCa cells promoting metastasis initiation.

## 1. Introduction

Prostate cancer (PCa) is one of the most prevalent cancer diseases in men around the world [1]. Between 20% and 30% of patients who originally respond to initial treatments can still develop androgen-independent and treatment-resistant bone metastasis, which is the main cause of death. Metastasis is a complex, multiple-step process led by intravasation of single cancer cells/clusters into vasculature from the primary site [2]. After circulating in blood flow and extravasation, only very few cancer cells that have homed and responded to the foreign microenvironment can eventually develop metastatic tumor growth [3]. More recently, a small subpopulation of cancer cells called cancer stem-like cells (CSCs) have been indicated to initiate PCa bone metastasis [4,5]. Those cells are characterized by high self-renewal capacity, tumorigenicity and chemoresistance. A number of proteins are identified as PCa CSC markers including CD44, integrin α2β1 and aldehyde dehydrogenase (ALDH) isoforms ALDH1A1 and ALDH7A1 [6,7,8]. Moreover, cell subpopulations with high ALDH activity have been reported as CSC-enriched subpopulations and characterized by high expression of CD44, integrin α2β1, enhanced clonogenicity in vitro and metastatic capacity in vivo [7]. Hence, targeting of this cell subpopulation may have a positive clinical outcome. Importantly, it is suggested that CSCs can be dedifferentiated from non-CSCs in multiple types of cancers including PCa [9,10,11]. This so-called CSC-plasticity is hypothetically driven by the molecular and cellular cues present in the tumor microenvironment [3,9]. Understanding the underlying mechanisms driving cancer plasticity is, therefore, essential for anti-CSC drug development. 

The emergence of CSCs are closely linked to the epithelial–mesenchymal transition (EMT) [12,13,14], a developmental program promoting cancer progression [15,16,17]. Once this happens, cancer cells lose cell–cell junction, gain motility and induce metastatic dissemination [15]. Multiple studies have indicated that the production of CSCs is partially a result of EMT [18]. Transient expression of EMT transcriptional factors Snail1 can induce tumor recurrence and metastasis [19,20]. In addition, EMT is a highly plastic process. Cancer cells can shift between EMT and its reversed program, mesenchymal to epithelial transition (MET), at different steps of metastatic cascades, endowing the cells with distinct properties like migration, invasion, quiescence and re-entry of tumor growth [21]. However, due to a lack of tools to detect single-cell status at each step of the metastatic cascade, the precise roles of EMT and MET in the whole metastatic process are still controversial [22]. 

Zebrafish (ZF) have been employed as a powerful platform for cancer research due to their high genomic conservation with humans [23,24,25]. They are widely used to model the initiation and malignant progression of different types of cancer [23,26,27]. ZF xenograft models with different human cancer cells have been established [25,28]. Due to an absence of mature adaptive immunity at the embryonic stage, human cancer cell lines and patient-derived primary cancer cells can survive, grow and metastasize in ZF [26]. Optical transparency of the embryo enables imaging of all steps of cancer progression at a single-cell resolution in the live animal. The high molecular similarity between human and ZF permits the human cancer cells to sense and respond to ZF microenvironment cues during cancer progression [29,30]. High-throughput screens can be easily performed to assess the metastatic capacity of different cancer cells and to test their therapeutic responses to drugs [27]. 

To visually and genetically trace PCa cell plasticity at the early stagy of metastasis, fluorescence-labeled PCa cells were intravenously injected into zebrafish, inducing formation of metastatic lesions in the zebrafish hematopoietic tissue. Immunofluorescence and RT-PCR analysis revealed an induction of EMT, enrichment of ALDH^hi^ CSC subpopulation and upregulation of CSC markers when the PCa cells colonized the metastatic site. We previously reported that Cripto is a stemness gene that governs PCa metastasis in zebrafish and mice xenografts [31]. Furthermore, we observed that co-culturing of PCa cells with osteoblasts induced Cripto expression, suggesting possible involvement of bone niche signals in its regulation [31]. Here, we showed that the expression of Cripto in the engrafted cells was upregulated by the ZF microenvironment. This microenvironment-induced Cripto drove experimental metastatic colonization through induction of EMT plasticity. Targeting of either Cripto or EMT transcriptional factors significantly inhibited metastatic growth. Altogether, our data indicated that human PCa cells can respond to zebrafish molecular and cellular cues, inducing Cripto-mediated EMT/CSC plasticity, which leads to metastatic tumor initiation.

## 2. Materials and Methods

### 2.1. Cell Culture

Human embryonic kidney cells HEK-293T (kindly provided by Dr. Sylvia Le Dévédec, Leiden Academic Center for Drug Research, Leiden University, Leiden, Netherlands) were maintained in DMEM supplemented with 10% FCS. Human PCa cell line PC-3-mCherry was maintained in Nutrient Mixture F-12K supplemented with 10% FCS, while PC-3M-Pro4-mCherry (kindly provided by Dr. Gabriel van der Pluijm, Department of Urology, Leiden University Medical Center, Leiden, Netherlands) was DMEM supplemented with 10% FCII (GE Healthcare Life Science, Pittsburgh, United States).

### 2.2. Zebrafish Maintenance, Tumor Cell Implantation and Metastasis Analysis

Wildtype zebrafish (ZF) line ABTL and transgenic line tg (Fli:GFP) [32] were handled in compliance with local animal welfare regulations and maintained according to standard protocols (www.ZFIN.org). Experiments were assessed by the Animal Experiments Committee (Dier Experimenten Commissie—DEC) and assessed by the Central Committee on Animal Testing (CCD).

Cancer cell transplantation was performed as described before [28]. In brief, at two days post-fertilization (dpf), dechorionated ZF embryos were anaesthetized with 0.003% tricaine (Sigma-Aldrich, Zwijndrecht, Netherlands) and plated on a Petri dish covered with 1.5% of solidified agarose. Cancer cells were trypsinized, suspended in PBS containing 2% polyvinylpyrrolidone (PVP; Sigma-Aldrich, Zwijndrecht, Netherlands) with a concentration of 200,000 cells/uL and loaded in into borosilicate glass capillary needles (1 mm O.D. × 0.78 mm I.D.; Harvard Apparatus, Holliston, MA, USA). Between 300 and 500 cancer cells labeled with either mCherry or Lifeact-mCherry were injected into the duct of Cuvier (DoC) of ZF embryos using a Pneumatic Picopump and a manipulator (WPI). The injected embryos were further maintained in a 34 °C incubator until the end of the experiments [28]. Images were acquired with a Leica M165 FC stereo fluorescent microscope at 1, 2, 4 and/or 6 d post injection (dpi). Intensity of mCherry fluorescence was further analyzed with image J software (IJ1.46r) and/or the ZF4 pixel counting program (Leiden Institute of Advanced Computer Science, Leiden University, Leiden, Netherlands). For high-resolution imaging, zebrafish embryos were placed on glass-bottom petri dishes and covered with 1% low melting agarose containing 0.003% tricaine (Sigma-Aldrich, Zwijndrecht, Netherlands). Images were acquired using a Leica SP8 confocal microscope (Leica Microsystems B.V, Amsterdam, Netherlands) and processed with image J software (IJ1.46r).

**Figure 1:**
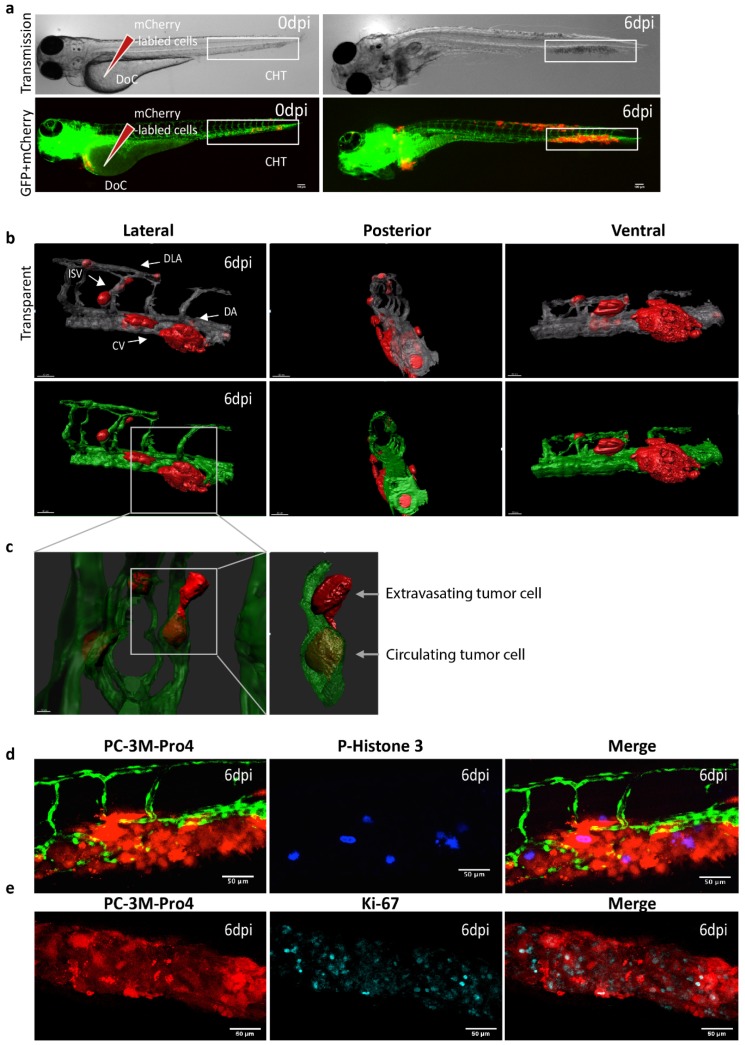
Intravenous transplantation of prostate cancer (PCa) cells induces extravasation and perivascular metastasis formation. (**a**) Schematic indication of cancer cell engraftment. PC-3M-Pro4-mCherry was injected into zebrafish (ZF) vasculature. Metastases was formed at 6 dpi. Red, cancer cells. Green, vessels. (**b**–**c**) High-resolution images were acquired using a Light-sheet microscope. Scale bar=100 um. (**b**) Three-dimensional overview of metastatic phenotype of the cancer cells at 6 dpi. Left, lateral. Middle, posterior. Right, Ventral. Up, solid vessels. Down, transparent vessels. Imaging was acquired using Zeiss Lightsheet Z1 at 40x magnification. Zeiss ZEN software was used for multiview fusion and deconvolution. Image J and Imaris 8.1 were applied for imaging stitching and 3D rendering. (**c**) High-magnification images to show single cell behaviors. Single cells were extravasated from intersegmental vessel (ISV), dorsal longitudinal anastomotic vessel (DLV) and Dorsal vein (DA). Up, solid vessels. Down, transparent vessels. (**d**–**e**) Whole-mount immunofluorescence against phosphorylated Histone 3 (**d**) and Ki-67 (**e**) at 6 dpi. Images were acquired using confocal. Scale bar = 50um.

For light-sheet microscopy, zebrafish embryos were anesthetized with 0.003% tricaine, embedded in 1% low melting agarose with tricaine and mounted in Zeiss light-sheet Z.1 capillary specimen holder, vertically immersed in imaging chamber containing water with tricaine. For whole-trunk imaging (Figure 1b) and for single-cell imaging (Figure 1c) 20× and 40× objectives were used, respectively. Specimens were imaged in single-track dual-channel mode to minimize spatial shift between channels. For each area, four stacks were acquired with specimen axial rotation of 90° between stacks. Specimen 3D structure was reconstructed using multiview fusion and deconvolution (Zeiss ZEN software (blue edition)). Surface rendering was performed using Imaris 8.1 with solid or semitransparent vasculature to aid observation of both intra- and extravascular cells.

### 2.3. Immunofluorescence

Whole-mount immunofluorescence on ZF was performed as described before [33]. ZF was fixed with 4% PFA, dehydrated and rehydrated with methanol in series concentration (25%, 50%, 75% and 100%). After permeabilization with 10ug/mL Protease K, embryos were blocked using blocking buffer containing 0.7%Triton X-100 and 5% sheep serum in 0.5% PBST. After incubation with primary (1/200) and fluorescence-conjugated secondary (1/200) antibodies, images were acquired using Leica SP8 confocal laser-scanning microscope. Antibodies used in the experiments included mouse anti-vimentin antibody (ab128507, Abcam, Cambridge, UK), rabbit anti-ki67 antibody (ab16667, Abcam Cambridge, UK) and rabbit anti-p-Histone3 (sc374669, Santa cruze, Dallas, TX, USA).

### 2.4. ALDEFLUOR Assay and Fluorescence-Activated Cell Sorting (FACS) Sorting

Cancer cells with high ALDH activity were detected and sorted using an ALDEFLUOR Assay kit (StemCell technology, Köln, Germany) following the manufacturer’s protocol. Briefly, 1–10 million PC-3M-Pro4-mCherry cells were treated with the ALDEFLUOR reagent. To set the gate for negative population, 500,000 PC-3M-Pro4-mCherry cells were treated with ALDEFLUOR reagent together with DEAB, an ALDH inhibitor. FACSCanto II (BD Biosciences, San Jose, CA, USA) was used for the measurement, and data were further analyzed with FCS Express Software (De Novo FCS Express 6). Each condition was independently repeated three times. For whole-mount ALDFLJUOR live staining on ZF, 6 dpf ZF was embedded in 1% low melting temperature agarose. Concentrated ALDEFLUOR reagent (10×) in ALDEFLUOR buffer was dropped on ZF. After incubation at 34 °C for 1 h, ZF was immediately imaged using a Leica SP8 confocal laser-scanning microscope.

### 2.5. RNA Extraction and qPCR

Total RNA isolation from ZF was performed as described before [33]. Metastasis samples in ZF were collected by cutting the whole metastatic site (80 fish per group) at 6 dpi using a micro dissection scissor (WPI, FL, USA). After cutting, the samples were immediately washed with cold PBS and stored in TRIzol (Sigma-Aldrich, Zwijndrecht, Netherlands) at −20 °C. The whole process was finished within 30 min. Whole RNA was isolated using the RNeasy mini kit (qiagen) following the manufacturer’s protocol. iScript™ cDNA Synthesis Kit (Bio-Rad, Utrecht, Netherlands) was used for cDNA synthesis and iQ™ SYBR® Green Supermix (Bio-Rad, Utrecht, Netherlands) was for qPCR as described in the manufacturer’s protocol. For each gene analysis, human specific primers were designed in order to measure the gene expression variation in human cancer cells. The species specificity was tested before the experiments. GAPDH was included as housekeeping for normalization. Three independent experiments were performed. 

### 2.6. Lentivirus Production and Transduction

Short hairpin RNA (shRNA) constructs against Cripto-1 (TRCN004890), Snail1 (TRCN0000063818, TRCN0000063821) and Zeb1 (TRCN0000017565, TRCN0000364631) were obtained from Sigma’s MISSION library (Kindly provided by the Department of Molecular Cell Biology, LUMC, Leiden, Netherlands). Lentiviruses were produced by transforming pLenti constructs, packaging plasmids psPAX2 and enveloped plasmids pMD2.G (Addgene, Watertown, MA, USA) into HEK-293T cells using lipoD293 (SignaGen Laboratories, Gaithersburg, MD, USA) as transforming reagent. Lentivirus supernatant was collected at 72 h after transformation. Cells were transduced with the lentiviruses using 6ug/mL Polybrene (Sigma-Aldrich, Zwijndrecht, Netherlands).

### 2.7. Statistics

Statistical analysis was performed with Graphpad Prism 7.0 (San Diego, CA, USA). A t-test was used to compare two groups and ANOVA for multiple groups. Data are presented as mean ±SEM or mean ±SD. p-values ≤0.05 are considered to be statistically significant (*p ≤ 0.05, **p < 0.01, ***p < 0.001, ****p < 0.0001)

## 3. Results

### 3.1. Intravenous Transplantation of PCa Cancer Cells into Zebrafish Leads to Development of Extravascular Metastatic Tumor Growth

Androgen-independent osteotropic PC-3M-Pro4-mCherry cells (300–500 cells) were intravenously injected into the duct of Cuvier (DoC) of tg(Fli:GFP) endothelial reporter transgenic zebrafish line with fluorescent vasculature at 2 d post fertilization (dpf) (Figure 1a) [32]. DoC is an open blood circulation channel connecting the heart and the trunk vasculature. Immediately after transplantation, cells hematogenously disseminated through the whole circulation. Most of the circulating cells regressed without extravasation and initiating tumor growth. However, exclusively at the posterior ventral end of caudal hematopoietic tissue (CHT), perivascular cells were able to extravasate and invade into tail fin within 1 d and developed perivascular metastatic lesions within 6 d (Figure 1a,b). CHT is a ZF hematopoietic organ at the early developmental stage with a certain molecular and cellular similarity to mice bone marrow [30,34]. To image cellular details of the metastatic phenotype, at 6 d post injection (dpi), high-resolution imaging was performed using a Light-sheet Confocal microscope (Figure 1b,c). This image proved that single cancer cells circulated in the blood flow and extravasated from intersegmental vessel (ISV), dorsal longitudinal anastomotic vessel (DLV), dorsal vein (DA) and caudal vein (CV) (Figure 1c). The metastatic tumor growth around CHT was characterized using immunofluorescence. Abundant phosphorylated Histone3-positive cells and Ki-67-positive cells were detected (Figure 1d,e), indicating that expended red fluorescent signal is indeed due to proliferation of PCa cells at the metastatic site. This novel experimental metastatic assay bypasses the primary tumor stage and intravasation but opens the possibility to image and study the mechanisms controlling metastatic initiation of PCa cells in a few days, instead of weeks, in rodent models. 

### 3.2. Oesteotropic PCa Cells with Enhanced EMT and CSC Traits Have Stronger Metastatic Potential in ZF

**Figure 2:**
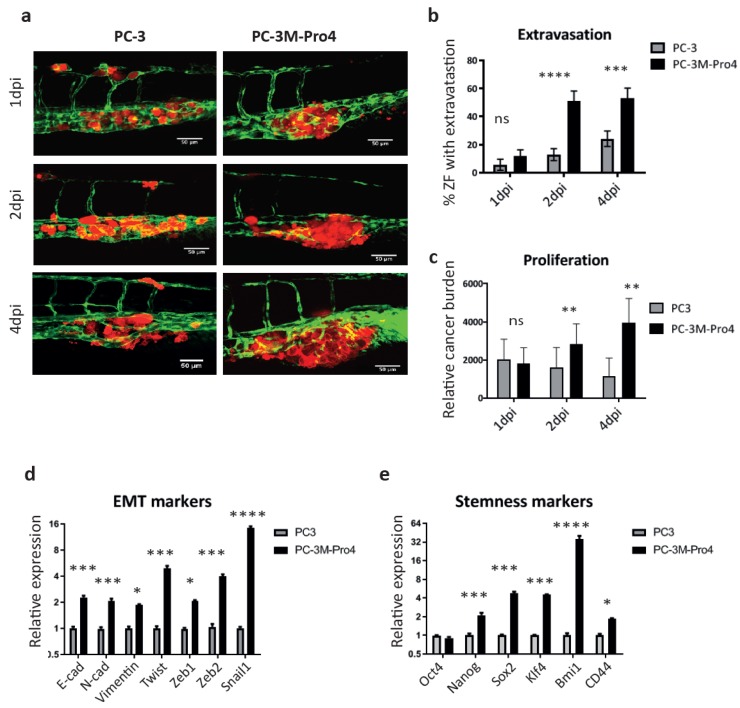
PC-3M-Pro4 with enhanced epithelial–mesenchymal transition (EMT) and cancer stem-like cell (CSC) traits in comparison with PC-3 have stronger extravasation and metastatic tumor-initiating capacities. (**a**) PC-3-mCherry and PC-3M-Pro4-mCherry were injected into ZF vasculature. Confocal images were acquired at the metastatic site at 1, 2 and 4 dpi. Green, vessels. Red, cancer cells. Scale bar=50um. (**b**–**c**) Extravasation and cancer cell burden at the metastatic site were analyzed. For extravasation analysis, % of ZF with more than 1 cell extravasated from the caudal vein and invaded into neighboring tissue was counted. For cancer cell burden quantification, total fluorescence of mCherry was measured using a ZF-4 pixel counting software. Experiment was independently repeated 2 times with 30 fish per group. Error bar presented as mean ±SEM. (**d**–**e**) The expression of EMT markers and stemness markers in PC-3 and PC-3M-Pro4 in culture was measured by qPCR. Experiment was independently repeated 3 times. Data were presented as mean ± SD.

To prove the usefulness of the ZF model, we compared the metastatic capacity of two PCa cell lines: PC-3 and PC-3M-Pro4. PC-3M-Pro4 is a metastatic subclone of PC-3, derived by four-fold orthotropic transplantation of PC-3 into mice prostate, endowing the cells with a strong bone metastatic potential in mice [35]. After intravascular injection into ZF embryos, PC-3 cells were circulating in blood flow at 1 and 2 d post injection (dpi) (Figure 2a). At 4 dpi, majority of the cells were cleared; however, a few cells survived and extravasated into the neighboring tissue around CHT (Figure 2a). In contrast, PC-3M-Pro4 cells docked at CHT at 1 dpi, extravasated and formed experimental metastasis at 4 dpi (Figure 2a). Extravasations of PC-3 and PC-3M-Pro4 were evaluated by counting the percentage of ZF with more than one cell extravasated from caudle vein and invaded the neighboring tissue (Figure 2a,b). PC-3 extravasated only in 5% of the engrafted ZF at 1 dpi, 10% at 2 dpi and 20% at 4 dpi, but PC-3M-Pro4 extravasated in 10% at 1 dpi and 50% at 2 and 4 dpi (Figure 2b). Metastatic tumor outgrowth was determined by measuring total fluorescence intensity in the trunk and CHT (Figure 2c). Total cancer cell burden of PC-3 at the metastatic site decreased over time, while the cancer cell burden of PC-3M-Pro4 gradually increased and was significantly higher at 2 and 4 dpi then PC-3 (Figure 2c).

Next, we questioned why PC-3M-Pro4 had stronger metastatic potential than PC-3. Gene signatures for EMT and cancer stemness were analyzed using qPCR. PC-3M-Pro4 cells in culture had elevated expression of mesenchymal markers N-cadherin, vimentin, twist, zeb1, zeb2 and snail1, and stemness markers Nanog, Sox2, Klf4, Bmi1 and CD44 (Figure 2d,e). Notably, PC-3M-Pro4 also exhibited a higher expression of epithelial marker E-cadherin, indicating these cells had a trait of partial EMT rather than full EMT where the expression of E-cadherin is missing. Those data suggest that enhanced metastatic capacity of PC-3M-Pro4 in zebrafish and mice models relies on the elevated expression of stemness and EMT genes.

### 3.3. Metastatic Outgrowth of PCa Cells in ZF is Associated with an Increase of EMT and Cancer Stemness Markers

**Figure 3:**
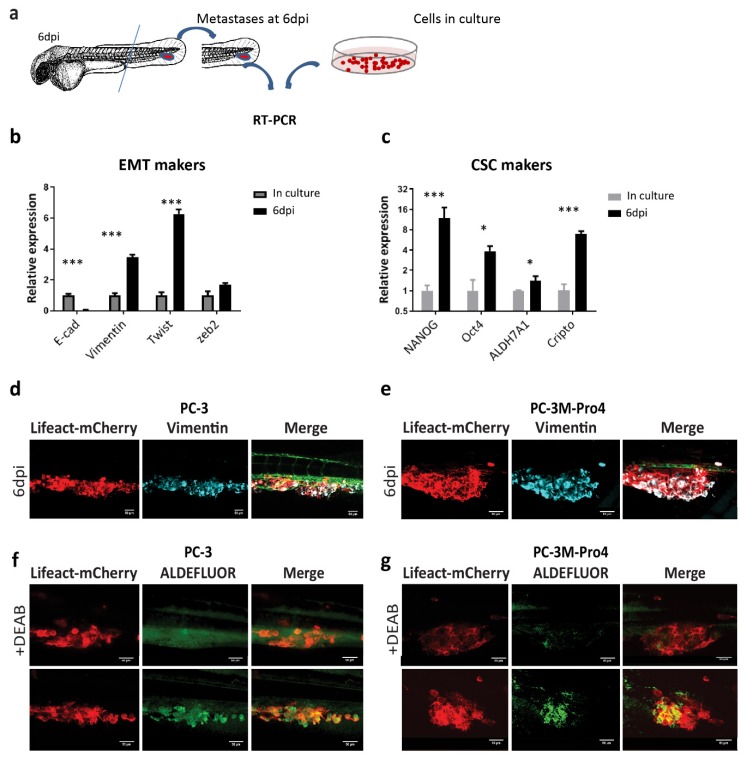
PCa cells obtain enhanced EMT and CSC traits after colonizing the ZF caudal hematopoietic tissue (CHT) area. (**a**) Schematic indication of RNA isolation from ZF metastases. At 6 dpi, ZF tails containing metastatic lesions were cut and collected for RNA isolation. (**b**–**c**) The expressions of EMT markers and stemness markers were compared in culture and in ZF metastases at 6 dpi. Experiments were independently repeated 3 times. Data were presented as mean ± SD (**d**–**e**) Immunofluorescence against vimentin on PC-3 and PC-3M-Pro4 in ZF metastases. (**f**–**g**) ZF engrafted with PC-3-lifeact-mCherry and PC-3M-Pro4-lifeact-mCherry was stained with ALDEFLUOR reagent with or without ALDH inhibitor DEAB at 4 dpi. Images were acquired using SP-8 confocal and processed with Fiji. Scale bar = 50um. Five fish were stained for each group.

We next measured how human cancer cells responded to the ZF microenvironment during the formation of metastatic lesions at the CHT area. At 6 dpi, RNA samples were collected from the metastatic lesions formed by PC-3M-Pro4 in 210 ZF embryos (Figure 3a). qPCR was performed using human specific primers without cross-reactivity with ZF tissue to compare gene expression profiles of the cancer cells in metastases and in cells culture Table S1. Consequently, the cancer cells from metastasis exhibited a significantly enhanced expression of mesenchymal marker vimentin, twist and zeb2 and a decrease of epithelial marker E-cadherin (Figure 3b). Moreover, these cells also gained expression of stemness genes including Nanog, Oct4, ALDH7A1 and Cripto (Figure 3c).

In addition, whole-mount immunofluorescence against vimentin was performed on PC-3 and PC-3M-Pro4 to further examine the EMT features. To visualize overlap between actin cytoskeleton and vimentin, we transduced PC-3 and PC-3M-Pro4 with Lifeact-mCherry, a small fluorescent peptide labelling actin filament. In vitro, all PC-3M-Pro4 cells had vimentin expression but only very few elongated cells in PC-3 expressed vimentin (data not shown). After colonizing ZF CHT at 6 dpi, however, both PC-3 and PC-3M-Pro4 displayed extensive and homogeneous expression of vimentin, indicating an acquisition of EMT features during the metastatic process (Figure 3d,e).

Given that the cancer cell subpopulation with high aldehyde dehydrogenase activity is identified in vitro as a CSC-enriched cell subpopulation, we applied ALDEFLUOR assay to measure the CSC features of cells at the metastatic site. At 4 dpi, live ZF engrafted with either PC-3 or PC-3M-Pro4 were stained with ALDEFLUOR reagent, a fluorescence dye labelling viable ALDH^hi^ cells. To set the negative control, some of the engrafted ZF were treated with the ALDH inhibitor DEAB when exposing to ALDEFLUOR reagent. Although no or very few ALDH signals were detected in the DEAB-treated ZF (Figure 3f,g), in DEAB-untreated ZF, both PC-3 and PC-3M-Pro4 exhibited strong ALDH signals at the metastatic site (Figure 3f,g). Altogether, our data reveal that both PCa cell lines respond to ZF microenvironment factors, resulting in an enhancement of EMT and CSC markers leading to metastatic tumor initiation.

### 3.4. Both ALDH^hi^ and ALDH^low^ Cells Obtain an Enhanced CSC Trait After Metastatic Colonization

**Figure 4:**
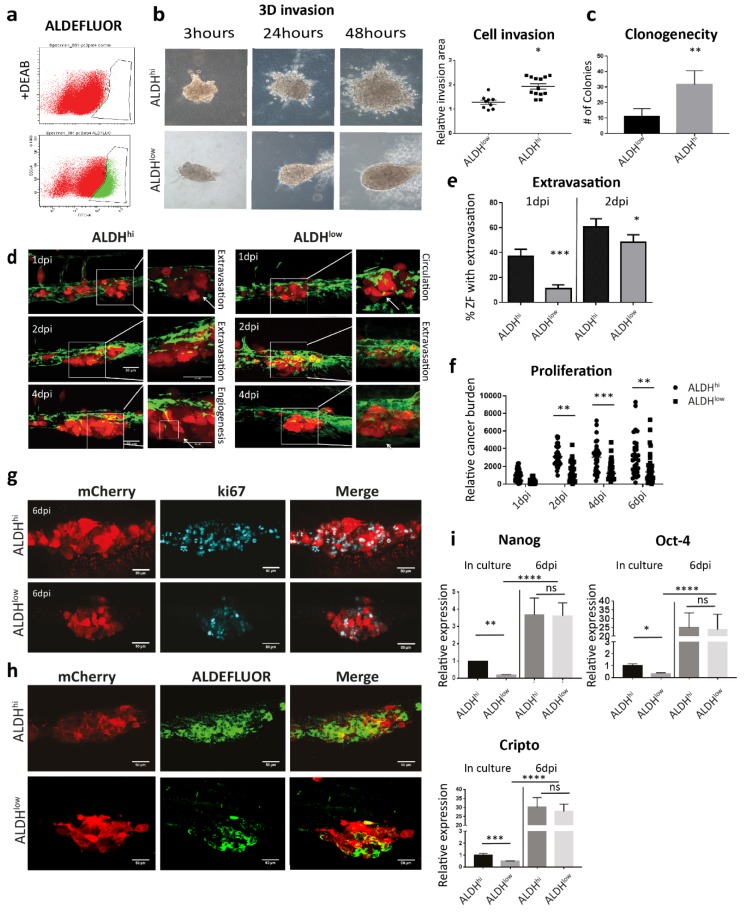
Both ALDH^hi^ and ALDH^low^ cell subpopulations obtain enhanced CSC traits after colonizing the ZF CHT area. (**a**) Gate setting for ALDH^hi^ and ALDH^low^ cell sorting. (**b**) After sorting, both ALDH^hi^ and ALDH^low^ subpopulations were respectively injected into type I collagen. Cell invasion was quantified by measuring the fold change of the invasive area after 48 h. Group size=10. Data were presented as mean ± SD. (**c**) ALDH^hi^ and ALDH^low^ cells were seed for clonogenicity. Number of colonies formed by the cells was counted after 14 d of culture. Ten spheres were measured for each group. Data were presented as mean ± SD. (**d**) ALDH^hi^ and ALDH^low^ cells were injected into ZF after sorting. Confocal images were acquired at 1, 2, 4 and 6 dpi. Scale bar = 50um. Green, vessel. Red, cancer cells. Left, 20X magnification. Right, 63X magnification. (**e**–**f**) Extravasation and cancer cell burden at the metastatic site were analyzed. Group size =30. Data were presented as mean ± SEM (**g**–**h**) Immunofluorescence against ki-67 and ALDEFLUOR staining were performed on engrafted ZF. Confocal images were acquired at the metastatic site. Scale bar = 50um. (**i**) Expression of stemness genes in ALDH^hi^ and ALDH^low^ was measured by qPCR in the cells after sorting and in the ZF metastases at 6 dpi. Experiments were independently repeated three times. Data were presented as mean ± SD.

Subsequently, we compared the metastatic potential of PCa CSCs and non-CSCs sorted by ALDEFLUOR assay from PC-3M-Pro4 (Figure 4a). The aggressiveness of the cells was firstly tested in vitro. 3D invasion assay was employed by embedding 500 cells into type-I collagen, and the size of the invasion area was measured after 48 h. In comparison with the ALDH^low^ cell, the ALDH^hi^ cells had a significantly enhanced invasive phenotype. (Figure 4b). Clonogenicity assay was employed to compare self-renewal capacity of the cells by seeding 200 single cells into 6-well plates. After 14 d of growth, significantly more colonies were formed by ALDH^hi^ cells (Figure 4c). Collectively, those in vitro data indicate that the ALDH^hi^ cells are more invasive and proliferative in vitro than its ALDH^low^ counterpart.

We next analyzed the aggressive phenotypes of ALDH^hi^ and ALDH^low^ cell subpopulations sorted from PC-3M-Pro4-mCherry in ZF xenografts. After engraftment, the ALDH^hi^ cells extravasated and invaded in 40% of the engrafted ZF at 1 dpi, while ALDH^low^ extravasated only in 10% of embryos (Figure 4d,e). At 2 dpi, the difference of extravasation between two cell types was smaller but still significant (Figure 4d,e). In addition, cancer cell burden at the metastatic site was measured. ALDH^hi^ cells had significantly higher cancer cell burden than ALDH^low^ (Figure 4f). Notably, at 6 dpi, some of the fish engrafted with ALDH^low^ still developed metastatic tumor growth; however, it was significantly lower compared to ALDHhi (Figure 4f). We showed by immunofluorescence that the metastases formed by both cell subpopulations were rapidly growing, since numerous Ki-67 (proliferation marker) positive cells were detected in both ALDH^hi^ and ALDH^low^ cells (Figure 4g).

To assess if the ZF microenvironment can regulate the stemness phenotype of the ALDH^hi^ and/or ALDH^low^ cells, ZF engrafted with either ALDH^hi^ or ALDH^low^ were stained with ALDFLUOR reagent at 4 dpi. Although extensive ALDEFLUOR signal was detected in the metastases formed by ALDH^hi^ cells (Figure 4h), some of the ALDH^low^ cells also displayed the positive ALDEFLUOR signal, indicating a re-enrichment of the ALDH^hi^ subpopulation in the engrafted ALDH^low^ cells during metastatic colonization (Figure 4h). 

Furthermore, we compared the expression of stemness genes in two cell subtypes directly after cell sorting and in metastatic lesions. Before injection, the ALDH^hi^ cells displayed enhanced expression of stemness markers Nanog, Oct4 and Cripto-1 than the ALDH^low^. Surprisingly, at 6 dpi after engraftment to ZF, the expressions of Nanog, Oct4 and Cripto were significantly increased to a similar level in both cell subtypes (Figure 4i). Taken together, our results indicate that both ALDH^hi^ and ALDH^low^ cells can respond to the microenvironment in ZF, inducing an augment of stemness feature.

### 3.5. Knockdown of Cripto Inhibited PCa Cell Metastatic Tumorigenicity Through Suppressing EMT Plasticity 

**Figure 5:**
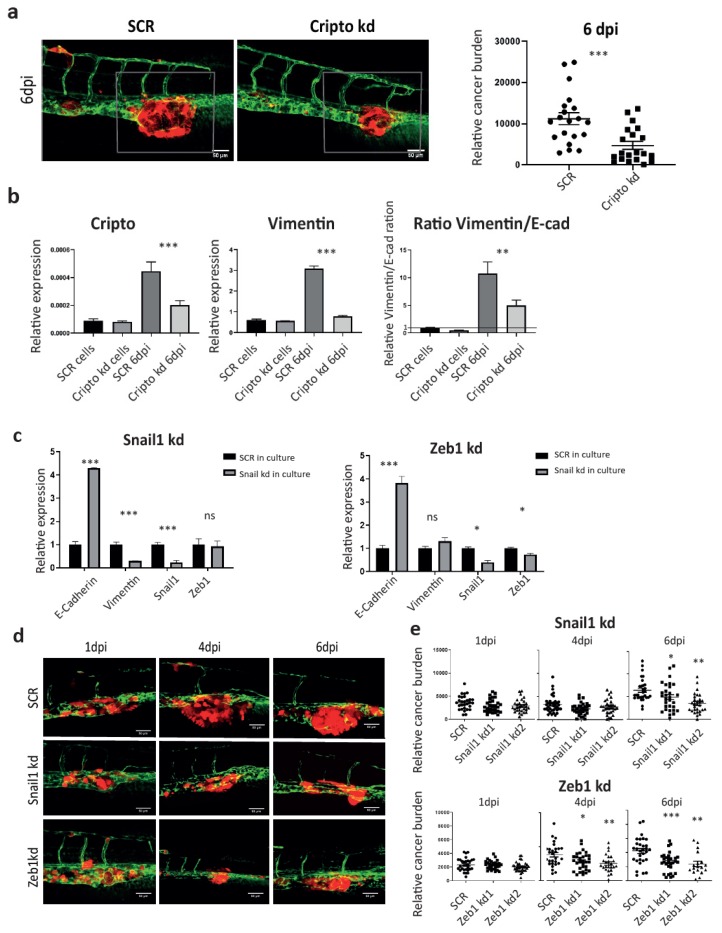
Cripto drives the metastatic phenotype through controlling EMT plasticity. (**a**) PC-3M-Pro4-mCherry-SCR and PC-3M-Pro4-mCherry Cripto kd were injected into ZF. Confocal images were acquired at 6 dpi. Green, vessels. Red, cancer cells. Scale bar = 50um. Cancer cell burden at the metastatic site was quantified by measuring total fluorescence intensity at 6 dpi. Group size = 30. (**b**) Expression of Cripto, stemness markers and EMT markers was measured in PC-3M-Pro4-mCherry-SCR and PC-3M-Pro4-mCherry-Cripto kd in culture and in ZF metastases at 6 dpi. Experiments were independently repeated three times. Data were presented as mean ± SD (**c**) Expression of EMT markers was compared between PC-3M-Pro4-mCherry-SCR, -Snail1 kd and -Zeb1 kd. Experiments were independently repeated three times. Data were presented as mean ± SD (**d**) PC-3M-Pro4-mCherry-SCR, -Snail1 kd and -Zeb1 kd were injected into ZF. Confocal images were acquired at 1, 4 and 6 dpi. Green, vessels. Red, cancer cells. Scale bar = 50um. (**e**) Cancer cell burden at the metastatic site was quantified by measuring total fluorescence intensity. Group size = 30. Data were presented as mean ± SEM.

The Nodal signaling co-receptor Cripto has been well documented to play an essential role in maintaining stemness in both embryonic stem cells and CSCs. In PCa, high expression of Cripto correlates with poor prognosis and high risk of metastasis in the patients [31]. We have previously shown that Cripto has a higher expression in ALDH^hi^ subpopulation. The knockdown of Cripto using shRNA significantly inhibited the size of ALDH^hi^ fraction in vitro and metastasis in ZF at 4 dpi and mice at 5 weeks after injection, respectively [31]. Here, we further investigated if microenvironment-induced Cripto was required for the cancer cell plasticity during metastasis (Figure 4i). To test this, PC-3M-Pro4 cells bearing either SCR or Cripto kd were engrafted into ZF. As expected, metastatic growth was significantly suppressed by Cripto kd at 6 dpi (Figure 5a). We collected the metastases tissue and performed cross-species RT-PCR using specific primers (Figure 5b). The knockdown of Cripto significantly reduced the vimentin/E-cadherin ratio in vivo, indicating that elevation of Cripto expression in engrafted cells by factors from the metastatic niche was indeed required for the acquisition of EMT traits during metastatic colonization (Figure 5b). 

We next investigated if metastatic growth in the ZF model was regulated by EMT. RNAi was therefore employed to target the EMT transcriptional factors Snail and Zeb1 to block the EMT trait. In vitro, the knockdown of Snail1 significantly inhibited vimentin expression but increased E-cadherin expression, while the knockdown of Zeb1 only enhanced E-cadherin expression without suppressing vimentin (Figure 5c). When both Snail kd and Zeb1 kd cells were respectively transplanted into ZF, total cancer burden at the metastatic site was significantly reduced at 6 dpi compared to the SCR control (Figure 5d), indicating that host microenvironment dependent acquisition of an EMT trait is essential for metastatic tumor initiation in ZF.

## 4. Discussion

Bone metastasis of PCa is a multiple-step process initiated by a few disseminated cancer cells. In this research, we established a zebrafish xenograft model to monitor the metastatic behavior of PCa cells at a single-cell resolution. We showed that human osteotropic, androgen-independent PCa cells can extravasate, proliferate and form perivascular metastases in 4–6 d. This metastatic behavior was associated with enhanced EMT and CSC traits in the cancer cells colonizing the zebrafish hematopoietic niche. Knockdown of Cripto, a Nodal co-receptor that was upregulated by ZF niche microenvironment in PCa metastasis, significantly inhibited metastatic tumorigenicity through suppression of EMT plasticity in ZF. In addition, targeting of EMT transcriptional factors in PCa cells prior engraftment also significantly suppressed metastatic growth at CHT. 

Our results indicate that the ZF microenvironment can induce human cancer cell plasticity. This cancer cell–microenvironment interaction in ZF resembles what happens in mammals [10,11,35]. In mice xenografts, human PCa cells can target the hematopoietic stem cell niche in bone marrow to establish a foothold inducing CSC enrichment. This CSC enrichment was partially regulated by osteoblasts [10,35]. When PCa cells were co-cultured with osteoblasts in vitro, the ALDH^hi^ subpopulation was enriched accompanied by an elevated expression of Cripto and induction of EMT, leading to an aggressive phenotype of the cancer cells. Given that the response of the human PCa cells to ZF CHT signals was similar to the PCa cell response to osteoblasts, the molecular cues driving Cripto expression and ALDH^hi^ subpopulation enrichment in both conditions seem to be comparable. 

Interaction between human cancer cells and ZF microenvironment was described before. CXCL-12, for instance, is an essential cytokine present in bone marrow leading to bone metastatic colonization of cancer cells [36]. In ZF, human cancer cells can sense host (ZF) CXCL-12, which is produced by mesenchymal stem cells in CHT [34], inducing metastatic colonization [29,30]. Targeting of either CXCR-4 (CXCL-12 receptor) in breast human cancer cells or CXCL-12 in ZF significantly inhibited extravasation and metastatic tumor growth at the CHT area. In addition, ZF myeloid cells were shown to guide human cancer cell extravasation and invasion by reorganizing extracellular matrix at the metastatic site [28]. Moreover, human cancer cells can comparably respond to the ZF microenvironment of ZF and mice comparably, inducing an activation of NF-ĸB–Activin A signaling axis which drives the metastatic CSC-like phenotype of the cancer cells [37]. Taken together, those studies indicated a functional interaction between human cancer cells and zebrafish microenvironment leading to the metastatic phenotype. A combination of different platforms including ZF xenografts, osteoblast co-culturing and mammalian xenografts can, therefore, be employed to study the molecular and cellular cues driving cancer cell plasticity.

In this research, we addressed how the ZF microenvironment governs metastatic onset of PCa cells. It was previously reported that Cripto has an enhanced expression in PCa bone metastasis and correlates with poor prognosis in stratified high-risk patients [31,38]. In PC-3M-Pro4, Cripto is upregulated in ALDH^hi^ subpopulation in culture and is required for experimental metastasis in zebrafish and mice xenografts [31]. Here, we showed that Cripto drives PCa cancer metastatic tumor initiation through induction of EMT at the metastatic onset. 

The role of EMT in cancer progression is still under discussion [21]. It was originally recognized as a driver of cancer cell invasion and metastatic dissemination [39]. After seeding to the niche, the EMT-reversed program MET is still required to initiate tumor outgrowth [39,40]. As evidence, metastatic tumor-initiating capacity was diminished after inducing sufficient, irreversible EMT by ectopic expression of EMT transcriptional factors (TFs), indicating the importance of MET in metastatic tumor outgrowth [40,41,42]. In contrast, other researches demonstrated that tumor initiation was associated with an occurrence of transient EMT, which endowed cancer cells with enhanced tumor-initiating capacity / CSC traits [19,20]. In prostate cancer, it was suggested that the cells with EMT status harbor CSC traits, while MET is associated with a more proliferative capacity [43]. Although this research proposed that the heterogenic cell population with a different EMT/MET status may have distinct functions in metastatic process, due to a limitation of animal models for real-time single cell tracking, they cannot directly monitor the dynamics of the cell states at the initial stage of metastatic tumor growth. Here, using ZF xenografts, we observed that both PC-3 and PC-3M-Pro4 obtained enhanced EMT and CSC traits after metastatic seeding. This transient EMT is essential for metastatic tumor initiation. Targeting of either Snail or Zeb1 to reverse EMT significantly inhibited metastatic tumorigenicity. To further elucidate how EMT-MET plasticity plays a role in long-term PCa metastatic tumor outgrowth, as described by others, studies with PCa cell lines bearing EMT reporter and inducible EMT/MET TFs expression systems can be performed using the immunodeficient ZF xenograft model [26]. This approach would allow to track single cancer cell EMT plasticity during whole metastatic cascade in long term.

Overall, in this study we employed a ZF xenograft model to track PCa cell plasticity at the early stage of metastasis. We showed that the PCa cells at the metastatic site obtained enhanced EMT and CSC traits, which were partially controlled by the induction of Cripto. Targeting either Cripto or other EMT factors may, therefore, have significant clinical potential. Moreover, our data highlight that ZF xenograft model can be applied as a powerful tool to study the role of cancer cell–microenvironment interactions in cancer stem- and EMT-plasticity regulation.

## Supplementary Materials

The following are available online at https://www.mdpi.com/2073-4409/9/4/797/s1, Table S1: Real time-qPCR primers.
