# Supplementary material for: Zebrafish Microenvironment Elevates EMT and CSC-Like Phenotype of Engrafted Prostate Cancer Cells
Source: Cells. 2020 Mar 26;9(4):797. doi: 10.3390/cells9040797 (PMC7226630; doi:10.3390/cells9040797)
Supplement: Supplementary file 1 [file cells-09-00797-s001.pdf]

Table 1. Real time-qPCR primers		
**Gene**	**Forwards primers**	**Reverse Primers**
CDH1	TTGACGCCGAGAGCTACAC	GACCGGTGCAATCTTCAAA
Vimentin	CCAAACTTTTCCTCCCTGAACC	CGTGATGCTGAGAAGTTTCGTTGA
Zeb1	CCATATTGAGCTGTTGCCGC	GCCCTTCCTTTCCTGTGTCA
Zeb2	GACCTGGCAGTGAAGGAAAA	GGCACTTGCAGAAACACAGA
Snail1	CCAGTGCCTCGACCACTATG	CTGCTGGAAGGTAAACTCTGGA
Twist	GCCGGAGACCTAGATGTCATT	TTTTAAAAGTGCGCCCCACG
CD44	TGGCACCCGCTATGTCCAG	GTAGCAGGGATTCTGTCTG
Nanog	AATACCTCAGCCTCCAGCAGATG	TGCGTCACACCATTGCTATTCTTC
OCT4	GAGAACCGAGTGAGAGGCAACC	CATAGTCGCTGCTTGATCGCTTG
Sox2	TACAGCATGTCCTACTCGCAG	GAGGAAGAGGTAACCACAGGG
Bmi1	TGATGTGTGTGCTTTGTGGAG	GGTCTGGTCTTGTGAACTTGG
Klf4	CCCAATTACCCATCCTTCCT	ACGATCGTCTTCCCCTCTTT
Cripto	CACGATGTGCGCAAAGAGAA	TGACCGTGCCAGCATTTACA
ALDH7A1	CAACGAGCCAATAGCAAGAG	GCATCGCCAATCTGTCTTAC
GAPDH	CTCTGCTCCTCCTGTTCGAC	GACCAAATCCGTTGACTCCG
